# A Positioning and Navigation Method Combining Multimotion Features Dead Reckoning with Acoustic Localization

**DOI:** 10.3390/s23249849

**Published:** 2023-12-15

**Authors:** Suqing Yan, Xiaoyue Xu, Xiaonan Luo, Jianming Xiao, Yuanfa Ji, Rongrong Wang

**Affiliations:** 1 Guangxi Key Laboratory of Precision Navigation Technology and Application, Guilin University of Electronic Technology, Guilin 541004, China; yansuqing@guet.edu.cn; 2 School of Information and Communication, Guilin University of Electronic Technology, Guilin 541004, China; 22022303147@mails.guet.edu.cn (X.X.); 1900200707@mails.guet.edu.cn (R.W.); 3 Guangxi Key Laboratory of Image and Graphic Intelligent Processing, Guilin University of Electronic Technology, Guilin 541004, China; 4 Department of Science and Engineering, Guilin University, Guilin 541006, China; xiaojianming@gxljc.edu.cn; 5 National & Local Joint Engineering Research Center of Satellite Navigation Localization and Location Service, Guilin 541004, China; jiyuanfa@guet.edu.cn; 6 GUET-Nanning E-Tech Research Institute Co., Ltd., Nanning 530031, China

**Keywords:** indoor positioning, dead reckoning, step length estimation, LASSO regression, extended Kalman filter

## Abstract

Accurate location information can offer huge commercial and social value and has become a key research topic. Acoustic-based positioning has high positioning accuracy, although some anomalies that affect the positioning performance arise. Inertia-assisted positioning has excellent autonomous characteristics, but its localization errors accumulate over time. To address these issues, we propose a novel positioning navigation system that integrates acoustic estimation and dead reckoning with a novel step-length model. First, the features that include acceleration peak-to-valley amplitude difference, walk frequency, variance of acceleration, mean acceleration, peak median, and valley median are extracted from the collected motion data. The previous three steps and the maximum and minimum values of the acceleration measurement at the current step are extracted to predict step length. Then, the LASSO regularization spatial constraint under the extracted features optimizes and solves for the accurate step length. The acoustic estimation is determined by a hybrid CHAN–Taylor algorithm. Finally, the location is determined using an extended Kalman filter (EKF) merged with the improved pedestrian dead reckoning (PDR) estimation and acoustic estimation. We conducted some comparative experiments in two different scenarios using two heterogeneous devices. The experimental results show that the proposed fusion positioning navigation method achieves 8~56.28 cm localization accuracy. The proposed method can significantly migrate the cumulative error of PDR and high-robustness localization under different experimental conditions.

## 1. Introduction

Location based services (LBS) have been involved in every aspect of people’s lives, such as obtaining location information about products in a shopping mall, searching for a vehicle in an underground parking lot, and monitoring the location of a patient in a hospital. At present, the global navigation satellite system (GNSS) can meet all LBS requirements in outdoor environments across all weather conditions and times [1,2]. However, in indoor environments, there are many interferences and obstacles that prevent satellite signals from entering indoor environments. Thus, the GNSS cannot support the location estimation [3]. Moreover, related studies have revealed that people currently spend more than 80% of their time in indoor environments. Overall, the study of LBSs in indoor navigation is of great research significance [4].

There are many common indoor localization methods, such as vision, Bluetooth, Wi-Fi, and positioning based acoustic. Vision-based positioning technology has good adaptability, but it is not able to protect the privacy issue well [5]. Bluetooth positioning is low- cost and requires simple implementation, but it is only suitable for positioning in a small range [6]. Wi-Fi positioning has a large coverage area and a wide deployment capacity with susceptible environmental interferences [7]. Currently, mobile smartphones are equipped with microphones and receivers for acoustic signals. Therefore, mobile smartphones can be used to send and receive acoustic signals, and no more additional infrastructure is required [8]. In addition, acoustic positioning has good security and there is no leakage of personal privacy information. Furthermore, acoustic signal positioning has the advantages of high accuracy and good compatibility [9,10]. However, acoustic signals are susceptible to environmental disturbances. These noises include inherent noises, the heating noises of electronic components, acoustic signal reflection, interference, diffraction, and pedestrian movement and conversations in the indoor environment.

Acoustic-based localization and navigation have become hot topics in current LBS research. Lopes, S.I., et al. [8] designed a passive time difference of arrival (TDOA) positioning system that is compatible with smartphones, which yielded a protocol for synchronizing acoustic beacons. Murakami, H., et al. [11] described a method for three-dimensional positioning using a smartphone with only an external speaker. Zhou, R.H., et al. [12] proposed a hybrid CHAN–Taylor algorithm. In this method, CHAN localization estimation is used as the initial value for the iteration of the Taylor algorithm. The Taylor iteration is interrupted when the error is below a preset threshold. The simulation results demonstrated that the hybrid CHAN–Taylor algorithm has better localization accuracy, convergence speed, and self-adaptability than the CHAN algorithm. Wang, X., et al. [13] used the combined CHAN–Taylor algorithm to effectively suppress non-line-of-sight (NLOS) errors for target localization in 3D indoor scenes. Yang, H.B., et al. [14] employed the hybrid CHAN–Taylor algorithm for underwater localization with a high accuracy of time-delay estimation. It was verified that the hybrid CHAN–Taylor algorithm can suppress the error of the CHAN algorithm.

Inertial measuring unit (IMU) navigation estimation includes an inertial navigation system (INS) and pedestrian dead reckoning (PDR) [15]. The INS estimates the current position with the angular velocity observed by the gyroscope sensors and the force observed by the accelerometer sensors. INS is not limited by application scenarios and is a very ideal navigation method. However, low-performance micro-electromechanical system (MEMS) devices are used and INS cannot provide reliable navigation results. PDR can provide long-term and stable relative positioning results by accurately detecting step counting and estimating step length and walking direction based on the movement characteristics of the pedestrian in the walking process [16,17,18]. It has simple implementation. Compared with the INS, PDR requires less accuracy for the sensor and enables better localization with limited cost. The implementation method has been widely used in the field of pedestrian navigation.

Many methods have been proposed to solve the problem of reducing the cumulative error of PDR. Yotsuya, K., et al. [19] presented an improvement to the accuracy of trajectories using a large amount of pedestrian trajectory data. Guo, S.L., et al. [20] presented a gait-detection method based on dual-frequency Butterworth filtering and a linear combination of multiple features combining the step frequency, the amplitude of acceleration, the mean of acceleration, and the variance of acceleration. Im, C., et al. [21] presented a multi-modal PDR system based on recurrent neural networks with a long short-term memory (LSTM) algorithm to extract potential features from sensor data. Yao, Y.B., et al. [22] proposed a method of identifying the step length based on the features extracted at each step, and the step-length error was approximately about 3%. Zhang, M., et al. [23] used adaptive step length estimation based on time windows and dynamic thresholds. Vathsangam, H., et al. [24] used Gaussian process-based regression (GPR) to estimate walking speeds and compared the performance of the Bayesian linear regression (BLR) and least squares regression methods. Zihajehzadeh, S., et al. [25] applied a linear model to estimate walking speed. Yan et al. [26] proposed an improved PDR method, which adds the previous three steps to predict the step value. The experimental results indicate that this method can obtain a more accurate step estimation.

Scholars have made some achievements in localization acoustic-based research. Reflections and diffraction from walls in indoor environments and noise from the environments can affect the accuracy performance of acoustic-based localization, and some outliers can even occur. The PDR not only has a low computational complexity but can also output accurate and reliable location information in a short period of time without relying on building any external infrastructure [27]. Nevertheless, cumulative errors in PDR can occur over time, which can have an extremely detrimental effect on the localization results [28,29].

To address the problems mentioned above, we propose a positioning and navigation system. To effectively mitigate the cumulative error of PDR, a novel step-length model with constraint LASSO regression [30,31] is proposed. This improved step-length model considers more relevant information to predict the current value than the state-of-the-art methods. The EKF is adopted to determine the target location by integrating acoustic-based localization with improved PDR. The main contributions of this paper are summarized below:

- **A novel weighted step-length model:** To improve the accuracy of step length, we propose a novel weighted step length with constraint LASSO regression in this paper. In the first step, the coarse current step length is predicted by combining the previous three steps inspired by Weinberg model. Then, the LASSO regression is used to correct the step estimation by combining the acceleration peak-to-valley amplitude difference, the walk frequency, the variance of acceleration, the mean acceleration, the peak median, and the valley median. The experimental results demonstrate that the proposed step-length model has better performance than the state-of-the-art methods.
- **A fusion positioning and navigation framework:** An EKF-based fusion positioning and navigation framework is presented. In this framework, the hybrid CHAN–Taylor method is used to estimate the location in the acoustic-based positioning. The improved PDR is adopted by the weighted step-length LASSO-based model. Then, the improved PDR is used as the state model and the acoustic estimation is used as the measurement model. The experiments show that the proposed positioning and navigation achieve better localization performance for different users, different devices, and different scenarios than existing methods. The framework is highly robust.

The rest of this paper is structured as follows. Section 2 provides related works about the current research. Section 3 introduces the positioning system methodology. The experimental results are depicted in Section 4. Finally, Section 5 summarizes the research in this paper.

## 2. Related Works

Fusion positioning technology has become a research hotspot in the field of indoor positioning. Song, X.Y., et al. [32] presented a method to validate the plausibility of PDR results using acoustic constraints between the acoustic source and the image source. Wang, M., et al. [33] proposed a method that combines the Hamming distance-based acoustic estimation with PDR. Yan proposed a CHAN–IPDR–ILS method in reference [34], which combines the CHAN algorithm and PDR algorithm. Al Mamun et al. [35] presented a lightweight fusion technique combining the PDR algorithm with the RSSI fingerprinting method. To decrease the cumulative error, landmarks are adopted to achieve localization. The experiment showed that the median positioning can reach 0.73 m. Poulose, A., et al. [36] proposed a fusion framework based on Wi-Fi and the PDR algorithm. The average localization accuracy of the combined position estimation algorithm was improved by 1.6 m compared with those of the separate algorithm. Lee, G.T., et al. [37] proposed a fusion algorithm based on Kalman filter (KF) for UWB localization and UWB-assisted PDR (U-PDR). Better performances were shown by comparing the UWB localization and PDR algorithm in the experimental results. Wu, J., et al. [38] proposed a text map-based indoor localization method that integrated RFID and the PDR method in a narrow corridor.

The EKF is a recursive algorithm that can be used for nonlinear systems and has a wide range of applications in the fields of navigation, positioning, and information fusion. Tian, X., et al. [39] used a two-step EKF iterative process to perform a state estimation of all the anchors in indoor environments. Yang, C.Y., et al. [40] constructed a 5G/geomagnetic/visual inertial odometry (VIO) positioning system based on an error-state EKF. Liu, W., et al. [41] proposed an autonomous navigation method combining EKF and a rapid exploration random tree (RRT) for four-wheel-steering vehicles to improve the accuracy of autonomous vehicle navigation in indoor environments. Mendoza, L.R., et al. [42] proposed a wearable ultrawideband indoor positioning system based on periodic EKF. Pak, J.M., et al. [43] proposed a switched extended Kalman filter bank (SEKFB) algorithm to overcome the problem of unstable noise covariance generated by isokinetic motion models for indoor localization.

Inspired by the existing positioning algorithms, we propose an indoor positioning method based on EKF fusion integrated with improved PDR and acoustic-based positioning. Specifically, in acoustic-based localization, a hybrid CHAN–Taylor algorithm is utilized to obtain the localization position. In PDR estimation, we propose a weighted fusion step improvement model based on LASSO. The step length estimation is obtained by the previous three steps and the Weinberg model. LASSO is used to modify the predicted step estimation, which makes the prediction value optimally close to the real value.

## 3. Methodology

In this section, we describe the EKF-based fusion localization architecture integrated into the acoustic-based and improved PDR positioning estimation. An overview of the proposed method is introduced in Section 3.1. The acoustic-based positioning method is described in Section 3.2. Step-count detection is presented in Section 3.3. The improved step model based on LASSO is proposed in Section 3.4. Section 3.5 depicts the heading direction calculation, and Section 3.6 analyzes the fusion method based on the EKF.

### 3.1. Overview

**Figure 1:**
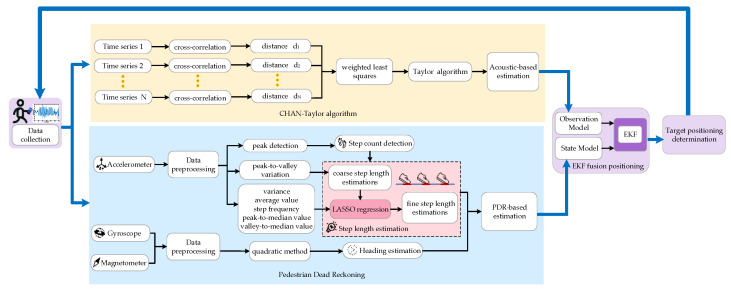
The methodological framework of the proposed positioning and navigation system.

The methodological framework of the proposed positioning and navigation system is presented in Figure 1. The framework is divided into four parts: data collection, acoustic-based estimation, PDR-based estimation, and EKF-based fusion positioning.

In data collection, acceleration, gyroscope, magnetometer, and ultrasonic signals can be sampled and saved in *.txt format in a smartphone. The collected data will be intermittently uploaded to the server terminal. After preprocessing the collected data, the estimation-based acoustic is solved by the hybrid CHAN–Taylor algorithm. Then, the peaks and valleys of accelerations are detected and step frequency can be determined. The coarse step-length estimation is obtained by the previous three steps and the maximum and minimum values of the acceleration at the current step, and then we combine LASSO regularization spatial constraint and the acceleration peak-to-valley amplitude difference, walking frequency, acceleration variance, mean acceleration, peak median, and valley median to achieve fine step-length estimation. The heading direction is obtained by quaternions method. In the target location estimation, the outliers are detected for acoustic-based estimation. Then, the EKF is used to fuse the target localization. The dead reckoning estimation is taken as the state vector, and the acoustic-based estimation is taken as the observation vector. Finally, the target location is obtained by incorporating the EKF method.

### 3.2. Acoustic-Based Estimation

**Figure 2:**
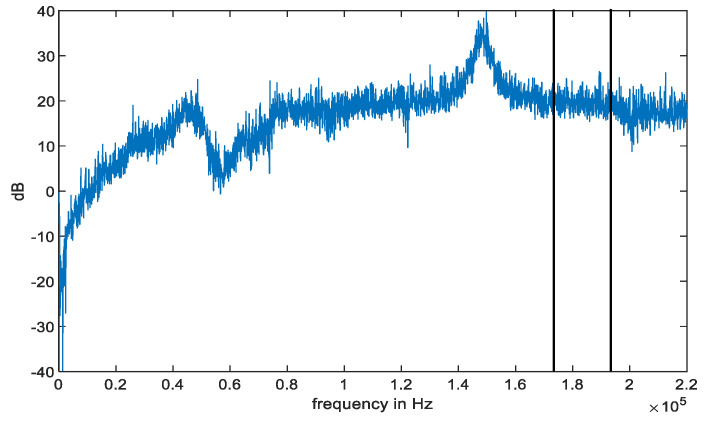
Spectrogram of the acoustic signal using a Vivo X30 smartphone.

Linear frequency modulation signals increase the transmission bandwidth of the signal by carrier frequency and perform pulse compression during reception. Additionally, linear frequency modulation signals have high resolution, can distinguish interference and targets at a distance, and can greatly simplify the signal processing system. A chirp is a typical nonstationary signal with great applications in sonar, radar, and other fields. In this paper, we use the chirp signal to transmit the acoustic signal. To validate the characteristics of the acoustic signals, we collect the acoustic signal using a Vivo X30 (Guangdong, China) smartphone. Collected signals are filtered and preprocessed through a Finite Impulse Response (FIR) bandpass filter, which basically filters out the interference information, such as indoor inherent noise and electronic components. In the final filtering stage, the adaptive minimum mean square error method is used to fuse the nonlinear approximation linearization, which once again alleviates the impact of noise. Figure 2 shows the strength fluctuation of the acoustic signal after filtering. From the figure, the acoustic signal is stable at 8–14 kHz and 17.5–19.5 kHz. Considering the interference of speech signals on positioning, pseudo ultrasound ranging from 17.5 to 19.5 kHz is selected as the acoustic localization source because the human ear is not sensitive to it. And the location estimation based on acoustic signal is solved using a cross correlation function. These data come from the same sending and receiving device every time. Device heterogeneity has little effect on the performance based on acoustic localization.

The CHAN algorithm is a non-iterative method with an analytic solution. The advantages of this algorithm are a high localization accuracy and low computation, but the localization accuracy is easily affected by complex indoor obstacles. The Taylor algorithm is a recursive algorithm that requires an initial position estimate. This algorithm solves the local least squares solution of the measurement error value at each recursion, continuously updating the estimate. The Taylor algorithm is robust and suitable for complex environments, but it is too dependent on initial values. This hybrid algorithm combines the advantages of the CHAN algorithm’s low computation and the Taylor algorithm’s good robustness. Therefore, for acoustic-based estimation, we chose the CHAN–Taylor hybrid algorithm for this paper. 

**Figure 3:**
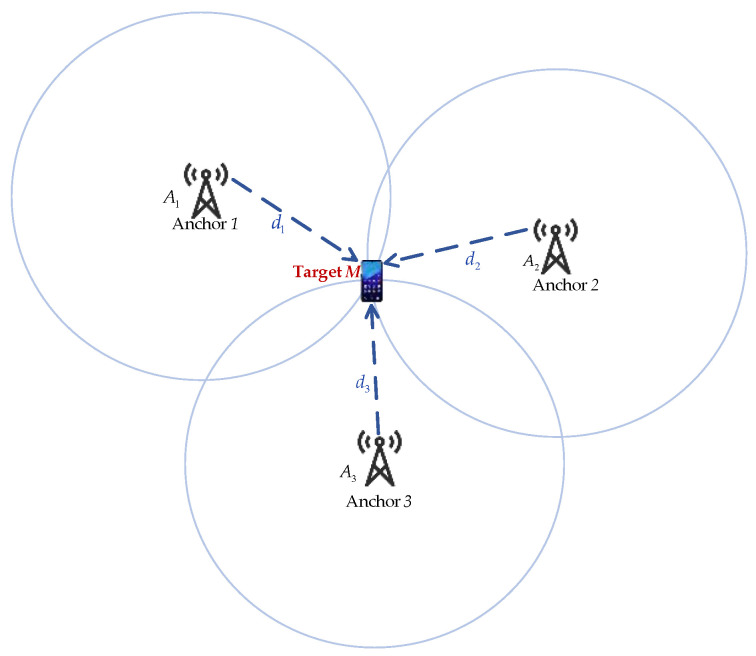
Spatial geometry distribution with three anchors $\left(A_{1},A_{2},A_{3}\right)$ and the target *M*.

The spatial geometric distribution of the three anchors and the target location is shown in Figure 3. Assuming the target location M is (x,y), the three anchors $A_{i}$ are $\left(x_{i},y_{i}\right)$, i=1,2,3.

The distance between the target M and the anchor $A_{i}$ is

(1) $$\sqrt{{\left(x_{i}-x\right)}^{2}+{\left(y_{i}-y\right)}^{2}}=d_{i}$$

where $d_{i}$ denotes the distance from the *i*-th anchor to the target M.

Expanding Equation (1), we can obtain

(2) $$u_{i}^{2}+x^{2}+y^{2}-2x_{i}x-2y_{i}y={\left(d_{i}\right)}^{2}$$

where $u_{i}^{2}={x_{i}}^{2}+{y_{i}}^{2}$.

Using anchor $A_{1}$ as the reference anchor, the difference $d_{i,1}$ between the *i*-th anchor and anchor $A_{1}$ can be derived:

(3) $$d_{i,1}=d_{i}-d_{1}=\sqrt{{\left(x_{i}-x\right)}^{2}+{\left(y_{i}-y\right)}^{2}}-\sqrt{{\left(x_{1}-x\right)}^{2}+{\left(y_{1}-y\right)}^{2}}$$

where $d_{1}$ denotes the distance from the first anchor to the target M.

Then,

(4) $$u_{i}^{2}+x^{2}+y^{2}-2x_{i}x-2y_{i}y=d_{i,1}^{2}+2d_{i,1}d_{1}+d_{1}^{2}$$

Letting ${x_{i,1}=(x}_{i}-x_{1})$,${y_{i,1}=(y}_{i}-y_{1})$,

(5) $$\frac{1}{2}[d_{i,1}^{2}-u_{i}^{2}+u_{1}^{2}]={-x}_{i,1}x-y_{i,1}y-d_{i,1}d_{1}$$

Suppose

$$H_{chan}=\frac{1}{2}\left[\begin{array}{c} d_{2,1}^{2}-u_{2}^{2}+u_{1}^{2}\\ d_{3,1}^{2}-u_{3}^{2}+u_{1}^{2}\\ ... \end{array}\right],\;G_{chan}=-\left[\begin{array}{ccc} x_{2,1} & y_{2,1} & d_{2,1}\\ x_{3,1} & y_{3,1} & d_{3,1}\\ ... & ... & ... \end{array}\right]\;\mathrm{and}\;Z_{chan}=\left[\begin{array}{c} x\\ y\\ d_{1} \end{array}\right].$$

Equation (5) can be expressed with matrix as follows:

(6) $$H_{chan}=G_{chan}Z_{chan}$$

Considering the measurement error, the error vector is depicted as

(7) $$e_{1}=H_{chan}-G_{chan}Z_{chan}^{o}$$

where $Z_{chan}^{o}$ is the value without Gaussian noise.

Its covariance matrix is

(8) $$\sigma =E[e_{1}e_{1}^{T}]=c^{2}BQB$$

where $B=diag\left\{d_{2},\;d_{3},\;d_{4},...,d_{N}\right\}$, and Q is the covariance matrix of the measurement errors.

The weighted least squares estimate of $Z_{chan}$ can be derived:

(9) $$Z_{chan}={\left(G_{chan}^{T}\sigma^{-1}G_{chan}\right)}^{-1}G_{chan}^{T}\sigma^{-1}H_{chan}$$

After obtaining the first estimate, the weighted squares method was again utilized to calculate the second estimate. The error variance can be expressed as

(10) $$e_{2}=H_{chan}^{\prime }-G_{chan}^{\prime }Z_{chan}^{\prime }$$

with the constraint:

(11) $$Z_{chan}^{\prime }=\left[\begin{array}{c} Z_{1}^{\prime }\\ Z_{2}^{\prime }\\ Z_{3}^{\prime } \end{array}\right]=\left[\begin{array}{c} x^{o}+\partial_{1}\\ y^{o}+\partial_{2}\\ d_{1}^{0}+\partial_{3} \end{array}\right]$$

where $\partial_{1},\partial_{2},\partial_{3}$ are the estimation errors. $Z_{chan}^{\prime }=\left[\begin{array}{c} ({x-x_{1})}^{2}\\ {\left(y-y_{1}\right)}^{2} \end{array}\right]$, $H_{chan}^{\prime }=\left[\begin{array}{c} {\left(Z_{1}^{\prime }-x_{1}\right)}^{2}\\ {\left(Z_{2}^{\prime }-y_{1}\right)}^{2}\\ {\left(Z_{3}^{\prime }\right)}^{2} \end{array}\right]$, $G_{chan}^{\prime }=\left[\begin{array}{cc} 1 & 0\\ 0 & 1\\ 1 & 1 \end{array}\right]$.

Then, the $Z_{chan}^{\prime }$ estimation is

(12) $$Z_{chan}^{\prime }={\left({\left(G_{chan}^{\prime }\right)}^{T}{\sigma \prime }^{-1}G_{chan}^{\prime }\right)}^{T}{\left(G_{chan}^{\prime }\right)}^{T}{\sigma \prime }^{-1}H_{chan}^{\prime }$$

where

(13) $$\sigma^{\prime }=4B^{\prime }Cov(Z_{chan})B^{\prime }$$

(14) $$B^{\prime }=diag\{x^{o}-x_{1},y^{o}-y_{1},d_{1}^{0}\}$$

The location of the target is

(15) $$\left[\begin{array}{c} x_{chan}\\ y_{chan} \end{array}\right]=\pm \sqrt{Z_{chan}^{\prime }}+\left[\begin{array}{c} x_{1}\\ y_{1} \end{array}\right]$$

Then, the estimate is used as the initial iterative solution of the Taylor algorithm. Specifically, the function $f\left(x_{i},y_{i},x,y\right)$ is assumed to represent the constraint relationship between the anchor and the target position. $f\left(x_{i},y_{i},x,y\right)$ is expanded in a Taylor series at $\left(x_{chan},y_{chan}\right)$, ignoring components above the second order to obtain the following equation:

(16) $$f\left(x_{i},y_{i},x,y\right)=f\left(x_{i},y_{i},x_{chan},y_{chan}\right)+\left(x-x_{chan}\right)f_{x_{i}}^{\prime }\left(x_{i},y_{i},x_{chan},y_{chan}\right)\;\;+(y-y_{chan})f_{y_{i}}^{\prime }\left(x_{i},y_{i},x_{chan},y_{chan}\right)$$

By defining ${∆x}_{c-t}=x-x_{chan}$ and ${∆y}_{c-t}=y-y_{chan}$, the following can be obtained:

(17) $$\begin{array}{cc} f\left(x_{i},y_{i},x,y\right)-f & \left(x_{i},y_{i},x_{chan},y_{chan}\right)\\ & ={∆x}_{c-t}f_{x_{i}}^{\prime }\left(x_{i},y_{i},x_{chan},y_{chan}\right)+{∆y}_{c-t}f_{y_{i}}^{\prime }\left(x_{i},y_{i},x_{chan},y_{chan}\right) \end{array}$$

According to Equation (3), $f\left(x_{i},y_{i},x_{chan},y_{chan}\right)$ can be represented as

(18) $$\begin{array}{cc} f\left(x_{i},y_{i},x_{chan},y_{chan}\right) & \\ = & \sqrt{{\left(x_{i}-x_{chan}\right)}^{2}+{\left(y_{i}-y_{chan}\right)}^{2}}-\sqrt{{\left(x_{1}-x_{chan}\right)}^{2}+{\left(y_{1}-y_{chan}\right)}^{2}} \end{array}$$

where $d_{i}^{chan}$ is the distance between the coordinate $\left(x_{chan},y_{chan}\right)$ and the anchor $A_{i}$.

Converting Equation (17) into matrix form is as follows:

(19) $$\varphi =H_{c-t}-G_{c-t}\sigma_{c-t}$$

where φ is the error vector and $H_{c-t}$ is the difference matrix between the real and measured values. $\sigma_{c-t}$ is the estimation error, as follows:

(20) $$H_{c-t}=\left[\begin{array}{c} d_{2,1}^{T}-(d_{2}-d_{1})\\ d_{3,1}^{T}-(d_{3}-d_{1})\\ \begin{array}{c} \ldots \\ d_{N,1}^{T}-(d_{N}-d_{1}) \end{array} \end{array}\right]$$

(21) $$G_{c-t}=\left[\begin{array}{cc} \frac{x_{1}-x_{chan}}{d_{1}}-\frac{x_{2}-x_{chan}}{d_{2}} & \frac{y_{1}-y_{chan}}{d_{1}}-\frac{y_{2}-y_{chan}}{d_{2}}\\ \frac{x_{1}-x_{chan}}{d_{1}}-\frac{x_{3}-x_{chan}}{d_{3}} & \frac{y_{1}-y_{chan}}{d_{1}}-\frac{y_{3}-y_{chan}}{d_{3}}\\ \begin{array}{c} \ldots \\ \frac{x_{1}-x_{chan}}{d_{1}}-\frac{x_{N}-x_{chan}}{d_{N}} \end{array} & \begin{array}{c} \ldots \\ \frac{y_{1}-y_{chan}}{d_{1}}-\frac{y_{N}-y_{chan}}{d_{N}} \end{array} \end{array}\right]$$

(22) $$\sigma_{c-t}=\left[\begin{array}{c} {∆x}_{c-t}\\ {∆y}_{c-t} \end{array}\right]$$

The weighted least squares solution is computed as

(23) $$\sigma_{c-t}={\left(G_{c-t}^{T}Q^{-1}G_{c-t}\right)}^{-1}G_{c-t}^{T}Q^{-1}H_{c-t}$$

In the next recursive operation, the iterative computation is performed after updating the coordinate values of the target estimate.

(24) $$\left\{\begin{array}{c} x_{chan-t}^{update}={∆x}_{c-t}+x_{chan}\\ y_{chan-t}^{update}={∆y}_{c-t}+y_{chan} \end{array}\right.$$

(25) $$\left\{\begin{array}{c} ∆x_{c-t}^{update}=x_{chan-t}^{update}-x_{chan}\\ ∆y_{c-t}^{update}=y_{chan-t}^{update}-y_{chan} \end{array}\right.$$

where $\left(x_{chan}^{update},y_{chan}^{update}\right)$ is the updated estimate calculated at each iteration. ${∆x}_{c-t}$ and ${∆y}_{c-t}$ are also constantly updated. The above process is repeated continuously until the iterative operation stops when the error meets the set conditions.

(26) $$|∆x_{c-t}^{update}|+|∆y_{c-t}^{update}|<\eta$$

where *η* is the error threshold.

Finally, the localization of the target *M* is determined as

(27) $$\left\{\begin{array}{c} x_{chan-t}=∆x_{c-t}^{update}+x_{chan}\\ y_{chan-t}=∆y_{c-t}^{update}+y_{chan} \end{array}\right.$$

### 3.3. Step Count Detection

During the data collection process, the collected data always include noise. Inaccurate step count detection, pseudo peaks and pseudo valleys, or missed detections will occur in the peak and valley detection if the original data are used. Therefore, noise cancellation processing is required for data collection.

**Figure 4:**
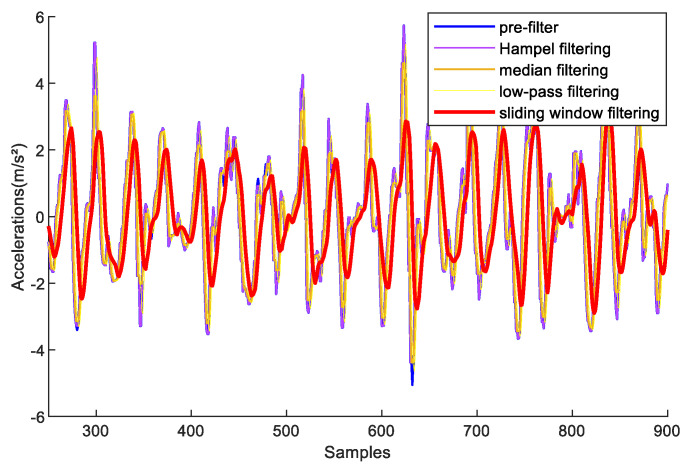
Comparison results among sliding-window filtering, low-pass filtering, median filtering, and Hampel filtering on acceleration processing.

Sliding-window filtering, low-pass filtering, median filtering, and Hampel filtering are common methods. To validate the performance of these methods, we recruited one volunteer to sample acceleration data in the experimental path at a stable speed. Figure 4 shows the acceleration results after filtering. The experiments demonstrate that sliding-window filtering retains better smoothness for the collected acceleration data than the other three methods. It has the best filtering performance compared with the other methods.

**Figure 5:**
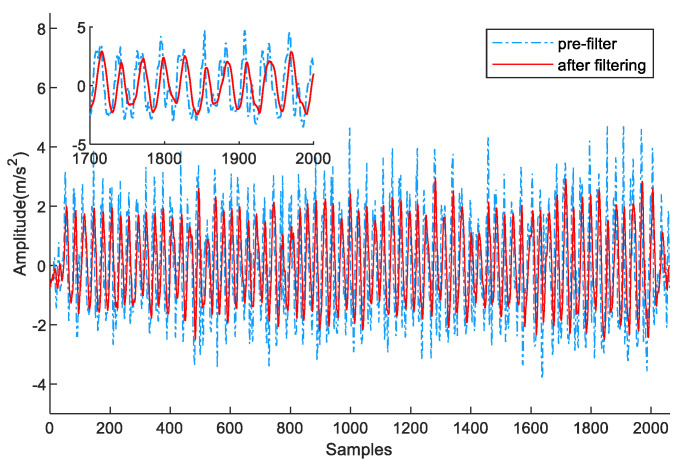
Acceleration data preprocessed by sliding-window filtering.

Therefore, we adopt sliding-window filtering to preprocess the original data. The width of the window size is chosen as 10 samples. In Figure 5, the original acceleration data are denoted by the blue dashed line, and the acceleration data after filtering are denoted by the red solid line. Compared with the original data, the filtered acceleration values have less fluctuation, which is favorable for step detection.

The peaks and valleys of the acceleration values are used to determine the step count. This mainly includes the following steps:

(1) Setting the acceleration threshold
  Different pedestrians have different motion patterns. Depending on the motion pattern, the acceleration threshold is set differently. When the acceleration value is greater than the preset threshold, this is determined as a candidate peak or a candidate valley.
(2) Setting the recognition sequence
  Acceleration exhibits a distinct regularity with successive peak–valley pairs. When one peak is recognized in the acceleration data, the valley will be judged in the next interval of data.
(3) Setting the time interval threshold
  The current candidate peak or candidate valley is valid only if the time interval between two neighboring peaks or valleys exceeds the preset time interval threshold.

**Figure 6:**
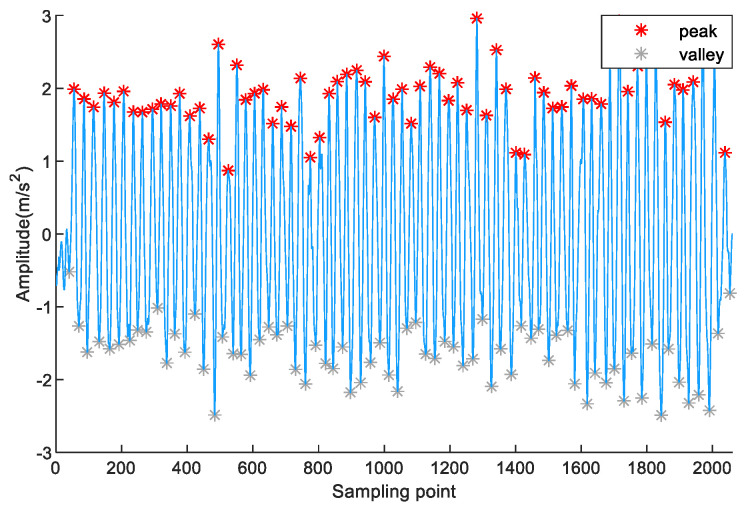
Peak and valley detection results on a 42 m experimental path.

To validate the above step detection method, a volunteer holding a Vivo X30 phone collected acceleration data on a 42 m experimental path. Figure 6 shows the maximum and minimum results of the pedestrian accelerations for each step on a 42 m experimental path. The maximum values of the pedestrian acceleration each step are marked in red stars, and the minimum values of the pedestrian acceleration each step are marked in gray stars. Therefore, step counts are accurately detected. This is because the above step detection methods can effectively identify pseudo-peaks and pseudo-valleys.

### 3.4. Step Length Prediction

Step-length prediction plays an important role in PDR localization. There are nonlinear and linear models in step-length prediction. A linear model only considers the relationship between step length and step frequency, which is not very accurate. A nonlinear model, which describes the more accurate correlation between the step size and motion parameters, is often used. The Scarlet model [44], Kim model [45], and Weinberg model [46] are typical nonlinear models. These three models are established on the basis of the relationship between the peak and valley of pedestrian acceleration and step length. However, pedestrian step length is related not only to the peak-to-valley amplitude difference in acceleration but also to multiple other potential characteristics. Therefore, it can achieve better performance when multiple characteristics are used to estimate the step length. Additionally, data overfitting and increased model complexity occur if there are too many characteristics.

Considering the continuity of adjacent steps and inspired by reference [26], the current step length is estimated by the weighted fusion of the previous three step lengths. In addition, to avoid overfitting, a regularization term constraining multiple characteristics is adopted to modify the step length. LASSO regression and ridge regression are commonly used regression methods with regularization terms. Ridge regression incorporates an L2 regularization term. LASSO regression incorporates an L1 regularization term and has an additional variable-filtering function compared with the former [47]. In addition, LASSO can not only prevent data overfitting but also reduces the model complexity. Therefore, LASSO regression is chosen to deal with the feature variables related to step length in this paper.

To address the above problems, we propose a novel step-length model; the coarse predicted value of the current step length is obtained using the weighted previous three steps based on the Weinberg model. The coarse step length ${SL}_{i}$ at time *i* can be obtained by the previous three steps and the acceleration maximum and minimum.

The coarse predicted step length ${SL}_{i}$ is described below:

(28) $${SL}_{i}=k_{1}*{SL}_{i-1}+k_{2}*{SL}_{i-2}+k_{3}*{SL}_{i-3}+k_{4}*K*\sqrt[{4}]{a_{i}^{max}-a_{i}^{min}}$$

with the LASSO constraint:

(29) $$min(\frac{1}{2}\sum_{i=1}^{M}{\left({SL}_{i}-\sum_{j=1}^{N}{{ACCF}_{j}^{i}\beta }_{j}\right)}^{2}+\lambda \sum_{j=1}^{N}\left|\beta_{j}\right|)$$

where ${SL}_{i-1}$, ${SL}_{i-2}$, and ${SL}_{i-3}$ are the lengths of the previous three steps. $k_{1}$, $k_{2}$, $k_{3},$ and $k_{4}$ are the weight factors. *K* is an empirical constant. $a_{i}^{max}{,\;a}_{i}^{min}$ are the maximum and minimum of the pedestrian accelerations for step *i*. M denotes the step number and *N* represents the number of features. ACCF is the six features of the acceleration values. $\beta =[\beta_{1},...,\beta_{N}]$ denotes the regression coefficient, and λ is the penalty coefficient, which is chosen based on 10-fold cross-validation.

Firstly, we can obtain the coarse step length ${SL}_{i}$ from Equation (28); ${SL}_{i}$ is used as the dependent variable of the model. The peak-to-valley amplitude difference, walking frequency, acceleration variance, acceleration mean, peak median, and valley median are extracted from the collected acceleration sensors. and the six motion features are used as the independent variables ACCF of the model. Then, we will find the optimal value from Equation (29).

(30) $$Loss=\frac{1}{2}\sum_{i=1}^{M}{\left({SL}_{i}-\sum_{j=1}^{N}{{ACCF}_{j}^{i}\beta }_{j}\right)}^{2}+\lambda \sum_{j=1}^{N}\left|\beta_{j}\right|$$

Equation (29) presents the minimum of loss function. The first part represents the squared loss function, and the second part represents the L1 regularization term. λ in Equation (29) adjusts the size of the regression coefficient $\beta_{j}$.

Expanding Equation (29), we can obtain the following:

(31) $$Loss=\frac{1}{2}\sum_{i=1}^{M}\left({{SL}_{i}}^{2}-2{SL}_{i}\sum_{j=1}^{N}{{ACCF}_{j}^{i}\beta }_{j}+{\left(\sum_{j=1}^{N}{{ACCF}_{j}^{i}\beta }_{j}\right)}^{2}\right)+\lambda \sum_{j=1}^{N}\left|\beta_{j}\right|$$

where ${ACCF}_{j}^{i}$ denotes the *i*-th sample value of the *j*-th feature variable.

To achieve better performance, the loss function in Equation (29) chooses the minimum value. Therefore, the first derivative of the regularization term in Equation (32) is expressed as follows:

(32) $$\frac{\partial \lambda \sum_{j=1}^{N}\left|\beta_{j}\right|}{\partial \beta_{j}}=\left\{\begin{array}{c} \lambda \beta_{j},\beta_{j}>0\\ 0,\beta_{j}=0\\ -\lambda \beta_{j},\beta_{j}=0 \end{array}\right.=\lambda sign(\beta_{j})$$

Then, the first derivative of Equation (27) is obtained:

(33) $$\frac{\partial Loss}{\partial \beta_{j}}=\frac{1}{2}\sum_{i=1}^{M}\left(-2{SL}_{i}{ACCF}_{j}^{i}+2{ACCF}_{j}^{i}(\sum_{j=1}^{N}{{ACCF}_{j}^{i}\beta }_{j})\right)+\lambda sign(\beta_{j})\;=\sum_{i=1}^{M}{\mathrm{ACCF}}_{j}^{i}(-{\mathrm{SL}}_{i}+(\sum_{j=1}^{N}{{\mathrm{ACCF}}_{j}^{i}\beta }_{j}))+\lambda sign(\beta_{j})$$

In the multidimensional derivative, the fixed values $\beta_{w}$ can be described as follows:

(34) $$\frac{\partial Loss}{\partial \beta_{w}}=\sum_{i=1}^{M}{\mathrm{ACCF}}_{j}^{i}(-{\mathrm{SL}}_{i}+(\sum_{j\neq w}^{N}{{\mathrm{ACCF}}_{j}^{i}\beta }_{j})+{x_{w}^{i}\beta }_{w})+\lambda sign(\beta_{w})\;=\sum_{i=1}^{M}{\mathrm{ACCF}}_{j}^{i}(-{\mathrm{SL}}_{i}+(\sum_{j\neq w}^{N}{{\mathrm{ACCF}}_{j}^{i}\beta }_{j}))+\sum_{i=1}^{M}{{ACCF}_{w}^{2i}\beta }_{w}+\lambda sign(\beta_{w})$$

Assuming that

(35) $$A_{j}=\sum_{i=1}^{M}{ACCF}_{j}^{i}(-{SL}_{i}+(\sum_{j\neq w}^{N}{{ACCF}_{j}^{i}\beta }_{j}))$$

(36) $$B_{j}=\sum_{i=1}^{M}x_{w}^{2i}$$

Equation (34) can be simplified as follows:

(37) $$A_{j}+B_{j}\beta_{w}+\lambda sign(\beta_{w})=0$$

Then, $\beta_{w}$ is

(38) $$\beta_{W}=\left\{\begin{array}{c} \frac{B_{j}+\lambda }{-A_{j}},\beta_{k}>0\\ 0,\beta_{k}=0\\ \frac{B_{j}-\lambda }{-A_{j}},\beta_{k}<0 \end{array}\right.$$

Finally, all regression coefficients are calculated. The final estimates of the step length are obtained:

(39) SL=ACCF·β+C

where C denotes the matrix of constants corresponding to the regression coefficients.

**Figure 7:**
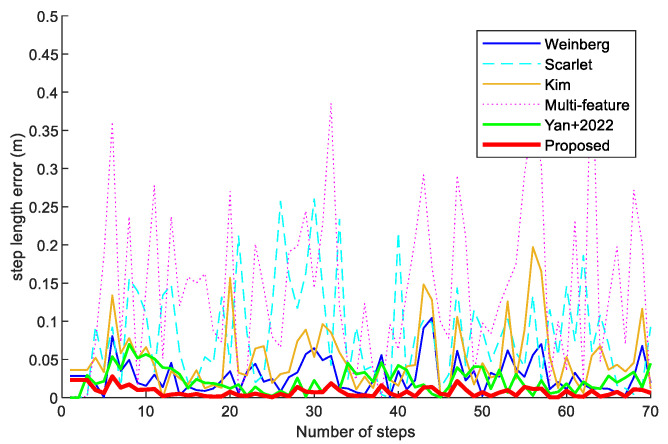
The step length error of the Weinberg, Scarlet, Kim, Multi-feature, Yan+ 2022 [26], and proposed step models.

To validate the weighted fusion step improvement model based on LASSO, a volunteer holding a Vivo X30 smartphone collected acceleration data along a 42 m experimental path. Figure 7 shows the step error of the Weinberg, Scarlet, Kim, Multi-feature, Yan+ 2022 [26], and proposed step models. From the results, the average step length error of the step improvement model proposed in this paper has the least errors compared with the others. Therefore, we can find that the step improvement method proposed in this paper is effective and the accuracy of the calculated step length is higher.

### 3.5. Heading Direction Calculation

Heading direction estimation is also an important factor in PDR and determines the direction of the entire track deflection [48]. The measured angular velocity of gyroscope sensors $\omega_{ib}^{b}$, the angular velocity of earth coordinate system relative to inertial coordinate system $\omega_{ie}^{b}$, the angular velocity of navigation coordinate system relative to earth coordinate system $\omega_{en}^{b},$ and the angular velocity of body coordinate system relative to navigation coordinate system $\omega_{nb}^{b}$ satisfy as follows:

(40) $$\omega_{ib}^{b}=\omega_{ie}^{b}+\omega_{en}^{b}+\omega_{nb}^{b}$$

(41) $$\left\{\begin{array}{c} \omega_{ie}^{b}=C_{n}^{b}\omega_{ie}^{n}=C_{n}^{b}C_{e}^{n}\omega_{ie}^{e}\\ \omega_{en}^{b}=C_{n}^{b}\omega_{en}^{n} \end{array}\right.$$

where $C_{e}^{n}$ is the transfer matrix between earth coordinate system and navigation coordinate system. $\omega_{ie}^{e}\;$is the angular velocity of earth coordinate system. $\omega_{en}^{n}$ is the angular velocity of navigation coordinate system relative to earth coordinate system.

The attitude angular velocity equation can be expressed in matrix as

(42) $$\omega_{nb}^{b}=\left[\begin{array}{c} \omega_{nbx}^{b}\\ \omega_{nby}^{b}\\ \omega_{nbz}^{b} \end{array}\right]=\left[\begin{array}{c} \omega_{ibx}^{b}\\ \omega_{iby}^{b}\\ \omega_{ibz}^{b} \end{array}\right]-C_{n}^{b}\left[\begin{array}{c} C_{13}\omega_{ie}^{e}+\omega_{enx}^{n}\\ C_{23}\omega_{ie}^{e}+\omega_{eny}^{n}\\ C_{33}\omega_{ie}^{e}+\omega_{enz}^{n} \end{array}\right]$$

where $C_{13}$, $C_{23}$, and $C_{33}$ are the transfer matrix vectors of earth coordinate system to navigation coordinate system, $C_{n}^{b}$ is the transfer matrix from navigation coordinate system to body coordinate system.

From Equation (42), we can obtain the angular velocity $\omega_{nb}^{b}$, and then we will continue to find the quaternion elements Q through the differential equation below:

(43) $$\dot{Q}=\frac{1}{2}Q\omega_{nb}^{n}$$

where ${Q=q}_{0}+q_{1}i+q_{2}j+q_{3}k$, $q_{0},q_{1},q_{2},q_{3\;}$are real numbers, and i, j, k are mutually orthogonal unit vectors. $\left|Q\right|=1$ is called a normalized quaternion.

Expanding Equation (43) into matrix as

(44) $$\left[\begin{array}{c} {\dot{q}}_{0}\\ {\dot{q}}_{1}\\ {\dot{q}}_{2}\\ {\dot{q}}_{3} \end{array}\right]=\frac{1}{2}\left[\begin{array}{cccc} 0 & -\omega_{nbx}^{b} & -\omega_{nby}^{b} & -\omega_{nbz}^{b}\\ \omega_{nbx}^{b} & 0 & \omega_{nbz}^{b} & -\omega_{nby}^{b}\\ \omega_{nby}^{b} & -\omega_{nbz}^{b} & 0 & \omega_{nbx}^{b}\\ \omega_{nbz}^{b} & \omega_{nby}^{b} & -\omega_{nbx}^{b} & 0 \end{array}\right]\left[\begin{array}{c} q_{0}\\ q_{1}\\ q_{2}\\ q_{3} \end{array}\right]$$

Once determining the vector ($q_{0},q_{1}{,q}_{2},q_{3}$), the attitude matrix can be depicted as follows:

(45) $$C_{b}^{n}=\left[\begin{array}{ccc} q_{0}^{2}+q_{1}^{2}-q_{2}^{2}-q_{3}^{2} & 2(q_{1}q_{2}-q_{0}q_{3}) & 2(q_{1}q_{3}+q_{0}q_{2})\\ 2(q_{1}q_{3}+q_{0}q_{3}) & q_{0}^{2}-q_{1}^{2}+q_{2}^{2}-q_{3}^{2} & 2(q_{2}q_{3}-q_{0}q_{1})\\ 2(q_{1}q_{3}-q_{0}q_{2}) & 2(q_{2}q_{3}+q_{0}q_{1}) & q_{0}^{2}-q_{1}^{2}+q_{2}^{2}-q_{3}^{2} \end{array}\right]$$

To simplify Equation (45), $C_{b}^{n}$ can be expressed as:

(46) $$C_{b}^{n}=\left[\begin{array}{ccc} C_{11} & C_{12} & C_{13}\\ C_{21} & C_{22} & C_{23}\\ C_{31} & C_{32} & C_{33} \end{array}\right]$$

The attitude directions are

(47) $$\begin{array}{c} \theta_{pitch\_b}=\arcsin C_{32}\\ \gamma_{roll\_b}=\left\{\begin{array}{ccc} \arctan\frac{-C_{31}}{C_{33}} & C_{33}>0 & \\ \left.\begin{array}{c} \arctan\frac{-C_{31}}{C_{33}}+\pi \\ \arctan\frac{-C_{31}}{C_{33}}-\pi \end{array}\right\} & C_{33}<0 & \left\{\begin{array}{c} \arctan\frac{-C_{31}}{C_{33}}<0\\ \arctan\frac{-C_{31}}{C_{33}}>0 \end{array}\right. \end{array}\right.\\ \psi_{head\_b}=\left\{\begin{array}{ccc} \left.\begin{array}{c} \arctan\frac{-C_{12}}{C_{22}}\\ \arctan\frac{-C_{12}}{C_{22}}+2\pi \end{array}\right\} & C_{22}<0 & \left\{\begin{array}{c} \arctan\frac{-C_{12}}{C_{22}}>0\\ \arctan\frac{-C_{12}}{C_{22}}<0 \end{array}\right.\\ \arctan\frac{-C_{12}}{C_{22}}+\pi & C_{22}<0 & \end{array}\right. \end{array}$$

### 3.6. EKF-Based Fusion Positioning

In fusion positioning, the acoustic-based estimation is set as the initial location of the target. To avoid the outliers, we set a threshold $D_{th}$ to detect anomalies in the estimation. At time *i* − 1, the acoustic-based estimation is ${Loc}_{i-1}^{chan-t}(x_{i-1}^{chan-t},y_{i-1}^{chan-t})$, and the estimation of the proposed dead reckoning method is ${Loc}_{i-1}^{p}(x_{i-1}^{p},y_{i-1}^{p})$.

**Case 1:** If the distance between the acoustic-based estimation and the localization is greater than the preset threshold $D_{th}$, the acoustic-based estimation is discarded as an outlier. Then, the estimation at time *i* − 1 is used for localization, where $\left(x_{i-1},y_{i-1}\right)={Loc}_{i-1}$.

**Case 2:** When the distance between the acoustic-based estimation and the localization is less than the preset threshold $D_{th}$, $\left(x_{i},y_{i}\right)$ is determined by EKF-based fusion positioning.

In our localization scheme, the PDR estimation is set as the state variable and the estimation is set as the observation variable. The state and observation vector are expressed as follows:

(48) $$X={\left[x_{pdr},y_{pdr},SL,\psi_{target}\right]}^{T}$$

(49) $$Z={\left[x_{chan-t},y_{chan-t},SL,\psi_{target}\right]}^{T}$$

where SL is the pedestrian step length and $\psi_{target}$ is the heading direction of the target. $\left(x_{pdr},y_{pdr}\right)$ is the PDR estimation, and $\left(x_{chan-t},y_{chan-t}\right)$ is the acoustic-based estimation.

In fusion localization, the observation equation and state equation of the EKF algorithm are described as follows:

(50) $$\left\{\begin{array}{c} X_{i}=F_{i-1}\left(X_{i-1}\right)+\omega_{i}\\ Z_{i}=\Psi_{i}\left(X_{i}\right)+\nu_{i} \end{array}\right.$$

where $i\in N=\left\{0,1,2,...\right\}$, $X_{i}\in R^{4}$ is the pedestrian target position to be estimated, which is the state vector of the Kalman filter. $Z_{i}\in R^{4}$ is the volume measurement vector, representing the acoustic estimate. $\omega_{i}\in R^{4}$ is the process noise. $\nu_{i}$ is the measurement noise, which satisfies a Gaussian distribution. $F_{i-1}\left(X_{i-1}\right)$ and $\Psi_{i}\left(X_{i}\right)$ are the nonlinear state and observation functions, respectively.

State vectors $X_{i}$, measure vectors $Z_{i}$ and noise signals $\omega_{i}$,$\nu_{i}$ satisfy statistical properties:

(51) $$Ε\left(\omega_{i}\right)=0,Ε\left(\upsilon_{i}\right)=0$$

(52) $$Ε\left(\omega_{i}\omega_{n}^{T}\right)=Q\left(i\right)\delta \left(i,n\right),i\;,n\in N$$

(53) $$Ε\left(v_{i}v_{n}^{T}\right)=R\left(i\right)\delta \left(i,n\right),\;i,n\in N$$

(54) $$Ε\left(\omega_{i}v_{n}^{T}\right)=0,i,\;n\in N$$

(55) $$Ε\left(X_{i}v_{n}^{T}\right)=Ε\left(X_{i}\omega_{n}^{T}\right)=Ε\left(Z_{i}v_{n}^{T}\right)=Ε\left(Z_{i}\omega_{n}^{T}\right)=0,i,\;n\in N$$

where $Q_{i}$ and $R_{i}$ are

(56) $$Q=\left[\begin{array}{cccc} \delta_{x}^{2} & 0 & 0 & 0\\ 0 & \delta_{y}^{2} & 0 & 0\\ 0 & 0 & \delta_{sL}^{2} & 0\\ 0 & 0 & 0 & \delta_{\psi }^{2} \end{array}\right]$$

(57) $$R=\left[\begin{array}{cccc} \delta_{x}^{\prime 2} & 0 & 0 & 0\\ 0 & \delta_{y}^{\prime 2} & 0 & 0\\ 0 & 0 & \delta_{sL}^{2} & 0\\ 0 & 0 & 0 & \delta_{\psi }^{2} \end{array}\right]$$

where $\delta_{x}^{2},\delta_{y}^{2}$ are the errors in PDR positioning. $\delta_{x}^{\prime 2},\delta_{y}^{\prime 2}$ are the errors in acoustic positioning. $\delta_{sL}^{2},\delta_{\psi }^{2}$ are the number of steps and direction angle of the PDR, respectively.

To estimate accurate pedestrian target location information, the nonlinear function needs to be linearized. The local linearization ${\hat{F}}_{i-1}$ and ${\hat{T}}_{i}$ of nonlinear functions $F_{i-1}$ and $T_{i}$ are expressed as follows:

(58) $$\left\{\begin{array}{c} {\hat{F}}_{i-1}={\left.{\left[\nabla_{X_{i-1}}F_{i-1}^{\prime }(X_{i-1})\right]}^{\prime }\right|}_{X_{i-1}={\hat{X}}_{i-1|i-1}}\\ {\hat{T}}_{i}={\left.{\left[\nabla_{X_{i}}\Psi_{i}^{\prime }(X_{i})\right]}^{\prime }\right|}_{X_{i}={\hat{X}}_{i|i-1}} \end{array}\right.$$

where

(59) $$\nabla_{X_{i-1}}=[\frac{\partial }{\partial X_{i-1}(1)},\frac{\partial }{\partial X_{i-1}(2)},\cdots ,\frac{\partial }{\partial X_{i-1}(N)}]\prime$$

The linearization of Equation (50) is described as follows:

(60) $$\left\{\begin{array}{c} X_{i}={\hat{F}}_{i-1}\cdot X_{i-1}+\omega_{i}\\ Z_{i}={\hat{\Psi }}_{i}\cdot X_{i}+\nu_{i} \end{array}\right.$$

Equation (60) can then be used to achieve fused localization using Kalman filtering. Thus, the fusion localization objective in this paper becomes the design of a suitable optimized filter for the system.

Design the Kalman recursive filter in the following form:

(61) $$\left\{\begin{array}{c} {\hat{X}}_{i|i-1}={\hat{F}}_{i}{\hat{X}}_{i-1}\\ \;\;\;\;\;\;\;\;\;\;{\hat{X}}_{i}={\hat{X}}_{i|i-1}+K_{i}(Z_{i}-{\hat{\Psi }}_{i}{\hat{X}}_{i|i-1}) \end{array}\right.$$

where $K_{i}$ is the filtering gain at moment i. ${\hat{X}}_{i}$ is the state estimate at moment *i* with initial value $X_{0}=X(0)$. ${\hat{X}}_{i}$ is the one-step state vector prediction at moment *i*.

In fusion localization, calculating the gain of the Kalman filter often requires calculating the inverse of a high-dimensional matrix, which increases the computational complexity. Therefore, it is necessary to consider suboptimal filters. To facilitate the analytical derivation of the suboptimization problem, the following two theorems are introduced.

**Theorem 1.** *For matrices A and B of appropriate dimensions, the trace of the matrix exists:*
(62) $$\left\{\begin{array}{c} \frac{\partial tr\left(AB\right)}{\partial A}=B^{T}\\ \frac{\partial tr\left(ABA^{T}\right)}{\partial A}=2AB \end{array}\right.$$

**Theorem 2.**  *The filter Equation (61) is estimated unbiased, implying that all* $i\in N=\left\{0,1,2,...\right\}$ *satisfies E{(i)} is zero.*

**Proof.** Combining Equations (60) and (61), the estimated value of the state vector ${\hat{X}}_{i}$ at time *i*:
(63) $$\begin{array}{cc} {\hat{X}}_{i} & ={\hat{X}}_{i|i-1}+K_{i}(Z_{i}-{\hat{\Psi }}_{i}{\hat{X}}_{i|i-1})\\ & ={\hat{X}}_{i|i-1}+K_{i}({\hat{\Psi }}_{i}X_{i}+v_{i}-{\hat{\Psi }}_{i}{\hat{X}}_{i|i-1})\\ & =(I-K_{i}{\hat{\Psi }}_{i})X_{i\left|i-1\right.}+K_{i}{\hat{\Psi }}_{i}X_{i}+K_{i}v_{i} \end{array}$$

Then the expectation of the state vector ${\hat{X}}_{i}$ is expressed as

(64) $$\begin{array}{cc} E[{\hat{X}}_{i}] & =E\left((I-K_{i}{\hat{\Psi }}_{i})X_{i\left|i-1\right.}+K_{i}{\hat{\Psi }}_{i}X_{i}+K_{i}v_{i}\right)\\ & =(I-K_{i}{\hat{\Psi }}_{i})E({\hat{X}}_{i|i-1})+K_{i}{\hat{\Psi }}_{i}E(X_{i})+K_{i}E(v_{i}) \end{array}$$

In the fusion localization process, the mean value of the time *i* = 0 is used as the estimated mean value, $\hat{X}(0)=E\{\bar{X}(0)\}$, E{X(0)}=0, $E({\hat{X}}_{i|i-1})=0$ According to Equation (51), $E\left\{v(i)\right\}=0.$

When $i\in N=\left\{0,1,2,...\right\}$, $E\{{\hat{X}}_{i}\}=0$. Thus, we have proved that the filter (53) is unbiasedly estimated. □

For the fusion localization in this paper, we need to solve the recursive filtering suboptimization problem. 

According to Equation (61), the estimation error is

(65) $$\begin{array}{cc} e_{i} & =X_{i}-{\hat{X}}_{i}=X_{i}-{\hat{X}}_{i|i-1}-K_{i}(Z_{i}-{\hat{\Psi }}_{i}{\hat{X}}_{i|i-1})\\ & \;\;\;\;\;=X_{i}-{\hat{X}}_{i|i-1}-K_{i}({\hat{\Psi }}_{i}X_{i}+v_{i}-{\hat{\Psi }}_{i}{\hat{X}}_{i|i-1})\\ & \;\;\;\;\;=(I-K_{i}{\hat{\Psi }}_{i})(X_{i}-{\hat{X}}_{i|i-1})-K_{i}v_{i}\; \end{array}$$

The mean square error of prediction is

(66) $$P_{i}=\left(e_{i}{e_{i}}^{T})=E({\langle (I-K_{i}{\hat{\Psi }}_{i})(X_{i}-{\hat{X}}_{i|i-1})-K_{i}v_{i}\rangle \langle (I-K_{i}{\hat{\Psi }}_{i})(X_{i}-{\hat{X}}_{i|i-1})-K_{i}v_{i}\rangle }^{T}\right)\;=E(\langle (I-K_{i}{\hat{\Psi }}_{i}){\hat{e}}_{i\left|i-1\right.}-K_{i}v_{i}\rangle \langle {\hat{e}}_{i\left|i-1\right.}^{T}(I-{{\hat{\Psi }}_{i}}^{T}K_{i}^{T})-v_{i}^{T}K_{i}^{T}\rangle )\;\;\;=E((I-K_{i}{\hat{\Psi }}_{i}){\hat{e}}_{i\left|i-1\right.}{\hat{e}}_{i\left|i-1\right.}^{T}(I-{{\hat{\Psi }}_{i}}^{T}K_{i}^{T})-(I-K_{i}{\hat{\Psi }}_{i}){\hat{e}}_{i\left|i-1\right.}v_{i}^{T}K_{i}^{T}\;{-K}_{i}v_{i}{\hat{e}}_{i\left|i-1\right.}^{T}(I-{{\hat{\Psi }}_{i}}^{T}K_{i}^{T})+K_{i}v_{i}v_{i}^{T}K_{i}^{T})$$

The measurement noise $\nu_{i}$ is uncorrelated with the one-step prediction error $e_{i\left|i-1\right.}$, resulting in

(67) $$E({{{\hat{e}}_{i\left|i-1\right.}v}_{i}}^{T})=E\{{\hat{e}}_{i\left|i-1\right.}\}E\{{v_{i}}^{T}\}=0$$

(68) $$E(v_{i}e_{i\left|i-1\right.}^{T})=E(v_{i})E(e_{i\left|i-1\right.}^{T})=0$$

Equation (66) can be expressed as

(69) $$\begin{array}{cc} P_{i} & =E\left\{(I-K_{i}{\hat{\Psi }}_{i}){\hat{e}}_{i\left|i-1\right.}{\hat{e}}_{i\left|i-1\right.}^{T}(I-{{\hat{\Psi }}_{i}}^{T}K_{i}^{T})+K_{i}v_{i}v_{i}^{T}K_{i}^{T}\right\}\\ & =(I-K_{i}{\hat{\Psi }}_{i})P_{i\left|i-1\right.}(I-{{\hat{\Psi }}_{i}}^{T}K_{i}^{T})+K_{i}R_{i}K_{i}^{T}\\ & =P_{i\left|i-1\right.}-P_{i\left|i-1\right.}{{\hat{\Psi }}_{i}}^{T}K_{i}^{T}-K_{i}{\hat{\Psi }}_{i}P_{i\left|i-1\right.}+K_{i}{\hat{\Psi }}_{i}P_{i\left|i-1\right.}{{\hat{\Psi }}_{i}}^{T}K_{i}^{T}+K_{i}R_{i}K_{i}^{T}\\ & =(I-K_{i}{\hat{\Psi }}_{i})P_{i\left|i-1\right.}{\left(I-K_{i}{\hat{\Psi }}_{i}\right)}^{T}+K_{i}R_{i}K_{i}^{T} \end{array}$$

Thus, the suboptimal problem for Equation (61) becomes solving Equation (69) to minimize the mean-square error, which is equal to the derivation of the matrix trace for Equation (69).

According to Theorem 1, the derivation of the matrix trace for Equation (69) is

(70) $$\frac{\partial tr(P_{i})}{\partial K_{i}}=-2{\left({\hat{\Psi }}_{i}P_{i\left|i-1\right.}\right)}^{T}+2K_{i}{\hat{\Psi }}_{i}P_{i\left|i-1\right.}{{\hat{\Psi }}_{i}}^{T}+2K_{i}R_{i}$$

To obtain *min* ($P_{i}$), we obtain

(71) $$K_{i}{\left({\hat{\Psi }}_{i}P_{i\left|i-1\right.}{{\hat{\Psi }}_{i}}^{T}+R_{i}\right)}_{i}=P_{i\left|i-1\right.}{{\hat{\Psi }}_{i}}^{T}$$

The filter gain is

(72) $$K_{i}=P_{i\left|i-1\right.}{{\hat{\Psi }}_{i}}^{T}{\left({\hat{\Psi }}_{i}P_{i\left|i-1\right.}{{\hat{\Psi }}_{i}}^{T}+R_{i}\right)}^{-1}$$

**Lemma 1.**  $X_{i}$ *is the position of the target to be estimated, which is a state vector of the extended Kalman filter.* ${\hat{X}}_{i|i-1}$ *is the one-step predicted value of the target, and *$\omega_{i}$ *is the process noise obeying a Gaussian distribution.* ${\hat{F}}_{i}$ *is an approximate linear state function. The one-step prediction estimation of the mean square error satisfies linear estimation with the mean square error of the previous moment.*

**Proof.** According to Equation (60), the mean square error of the one-step prediction estimate is
(73) $$P_{i\left|i-1\right.}=E\{{\hat{X}}_{i|i-1}{\hat{X}}_{i\left|i-1\right.}^{T}\}\;=E\{({\hat{F}}_{i-1}{\hat{X}}_{i-1}+\omega_{i})({\hat{F}}_{i-1}{\hat{X}}_{i-1}+\omega_{i}{)}^{T}\}\;=E({\hat{F}}_{i-1}{\hat{X}}_{i-1}{\hat{X}}_{i-1}^{T}{\hat{F}}_{i-1}^{T})+E({\hat{F}}_{i-1}{\hat{X}}_{i-1}{\omega_{i}}^{T})+E(\omega_{i}{\hat{X}}_{i-1}^{T}{\hat{F}}_{i-1})+E(\omega_{i}{\omega_{i}}^{T})$$

According to Equation (65), we obtain

(74) $$E{(\hat{F}}_{i-1}{\hat{X}}_{i-1}{\hat{X}}_{i-1}^{T}{\hat{F}}_{i-1}^{T})={\hat{F}}_{i-1}E({\hat{X}}_{i-1}{\hat{X}}_{i-1}^{T}){\hat{F}}_{i-1}^{T}\;={\hat{F}}_{i-1}P_{i\left|i-1\right.}{\hat{F}}_{i-1}^{T}$$

Compute the second and third terms of Equation (73), respectively.

(75) $$E({\hat{F}}_{i-1}{\hat{X}}_{i-1}{\omega_{i}}^{T})={\hat{F}}_{i-1}E({\hat{X}}_{i-1}{\omega_{i}}^{T})={\hat{F}}_{i-1}E({\hat{X}}_{i-1})E({\omega_{i}}^{T})$$

(76) $$E(\omega_{i}{\hat{X}}_{i-1}^{T}{\hat{F}}_{i-1})=E(\omega_{i}{\hat{X}}_{i-1}^{T}){\hat{F}}_{i-1}$$

Based on Equations (52) and (54) of the previous fusion localization model, the following can be obtained:

(77) $$E(\omega_{i}{\hat{X}}_{i-1}^{T}{\hat{F}}_{i-1})=E\left({\hat{F}}_{i-1}{\hat{X}}_{i-1}{\omega_{i}}^{T}\right)=0$$

(78) $$E(\omega_{i}{\omega_{i}}^{T})=Q_{i}$$

The one-step prediction mean square error is

(79) $$P_{i\left|i-1\right.}={\hat{F}}_{i-1}P_{i-1}{\hat{F}}_{i-1}+Q_{i}$$

Thus, the mean square error one-step prediction value is proved. □

Substituting Equation (79) into (72), the filter gain $K_{i}$ at moment i can be derived based on the minimum mean square error. The minimum mean square error under suboptimal filtering is obtained by substituting the $K_{i}$ obtained from the projection into Equation (69). Thus, the suboptimal estimation problem of fusion localization is solved.

The filter gain design in Equation (72) does not require a very large dimensional inversion of the inverse. A fusion localization scheme is established based on Theorems 1, 2, and Lemma 1. In this paper, the focus is on the transient characteristics, where the filtered mean-square error is obtained at each sampling instant i. The appropriate gain is designed to make the fusion localization sub-optimal.

## 4. Results

In this section, the experimental setup is depicted in Section 4.1. Then, the localization accuracy of the LASSO-based weighted fusion step improvement model is analyzed in Section 4.2. In Section 4.3, the CDF positioning performance of the EKF-based PDR combined with the acoustic estimation method is reported. The mean and RMS error performance of the EKF-based PDR combined with the acoustic estimation method is discussed.

### 4.1. Experimental Setup

**Figure 8:**
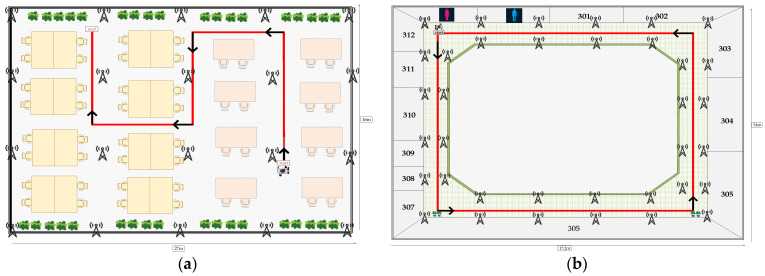
Floor plan of the experimental site: (**a**) scene 1, (**b**) scene 2.

In this paper, we conducted experiments in two indoor environments. Scenario 1, with dimensions of 27 × 16 × 3 m^3^, is a reading room, similar to a seminar room. There are many tables, some air conditioners, and potted plants in the reading room. The windows in the reading room are made of glass on both sides and walls on the other sides, which may affect signal reflection and absorption. The second scene, at 34 × 17.3 × 3 m^3^, is a big, closed corridor that follows an indoor loop. This scene is more open compared with the first scene. The anchor distribution is shown in Figure 8. Twenty-five beacons are deployed in the first experimental scenario and thirty-six beacons in the second scenario. The red solid line denotes the pedestrian movement trajectory, and the black solid arrows are the direction of movement. In Figure 8a, the experimental path is along the desks in the reading room. The start and end points are not the same location. In Figure 8b, the experimental path is a closed rectangle with the same start and endpoints.

In this experiment, we invited a female volunteer, 160 cm in height (#1), and a male volunteer, 181 cm in height (#2), to collect acoustic signals and IMU data. The two volunteers, holding Vivo X30 and OPPO K5 smartphones, walked along the test path with a 0.6 m/step speed several times, respectively.

### 4.2. Improved Step Length Performance

To assess the performance of the weighted fusion step estimation model based on LASSO, we conducted experiments on the Scarlet model, Kim model, Weinberg model, multifeatured model, Yan+ 2022 [26] model, and our model.

**Figure 9:**
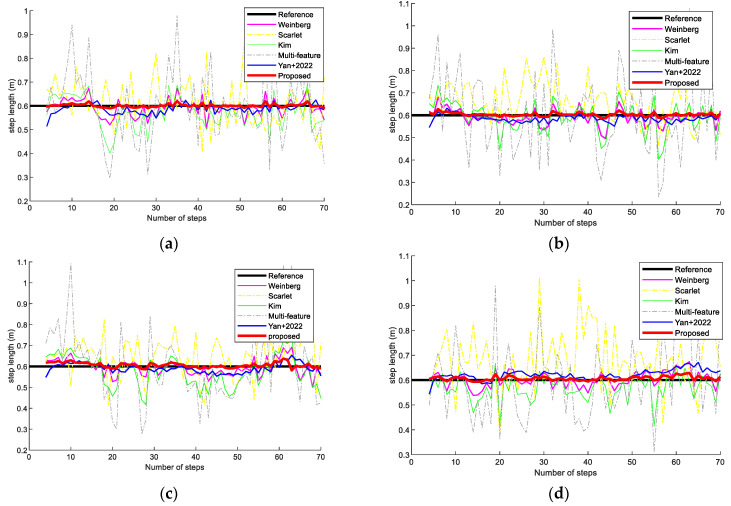
Step-length estimation comparison on different methods at scene 1. (**a**) Volunteer #1 using an OPPO K5 smartphone. (**b**) Volunteer #1 using a Vivo X30 smartphone. (**c**) Volunteer #2 using an OPPO K5 smartphone. (**d**) Volunteer #2 using a Vivo X30 smartphone.

Figure 9 shows the step-length results using a Vivo X30 and an OPPO K5 smartphone (Guangdong, China) for two volunteers, in scene 1, respectively. The proposed step estimation model is more accurate and produced a result closer to the real step length than the state-of-the-art step length models for different volunteers and mobile devices in scene 1. This is because the step estimation model proposed in this paper considers not only the first three steps but also the acceleration peak-to-valley amplitude difference, walk frequency, variance of acceleration, mean acceleration, peak median, and valley median. The method can supply more features to predict step length and can effectively mitigate the errors in the approximate symmetry.

**Figure 10:**
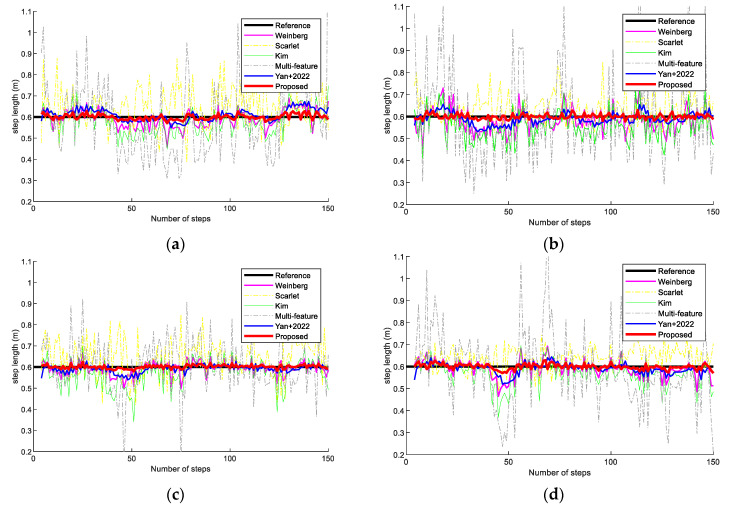
Step-length comparison on different methods in scene 2. (**a**) Volunteer #1 using an OPPO K5 smartphone. (**b**) Volunteer #1 using a Vivo X30 smartphone. (**c**) Volunteer #2 using an OPPO K5 smartphone. (**d**) Volunteer #2 using a Vivo X30 smartphone.

Figure 10 presents step-length results with two volunteers holding the Vivo X30 and OPPO K5 smartphones in scene 2. The experimental results show that the proposed step-length improvement model also performs better than the state-of-the-art step-length models. This is because the proposed step-length model has better robustness and can avoid the effects of different pedestrians and devices.

**Figure 11:**
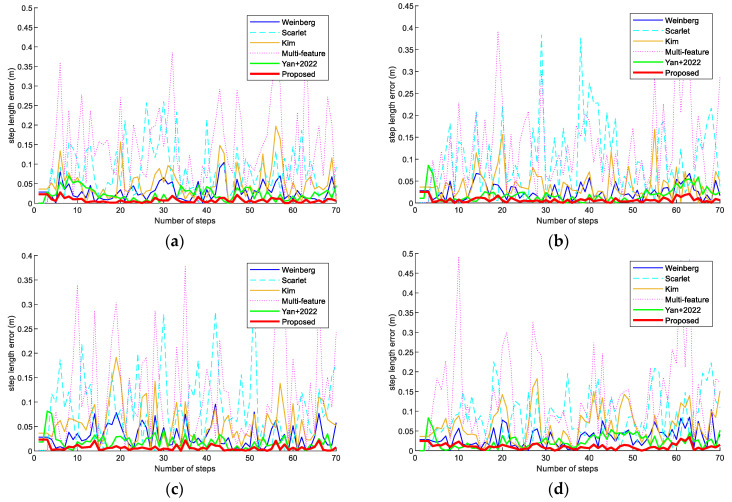
Step-length errors of the Weinberg, Scarlet, Kim, Multi-feature, Yan+ 2022 [26], and proposed step models in scene 1. (**a**) Volunteer #1 using an OPPO K5 smartphone. (**b**) Volunteer #1 using a Vivo X30 smartphone. (**c**) Volunteer #2 using an OPPO K5 smartphone. (**d**) Volunteer #2 using a Vivo X30 smartphone.

In addition, we compared the step errors of the Weinberg, Scarlet, Kim, Multi-feature, Yan+ 2022 [26], and proposed step models in scene 1. In Figure 11, it can be observed that the step errors of the step-length model proposed in this paper are smaller than those of the other models. The reason is that the proposed step-length estimation model combines various influencing features to estimate the step length in a more comprehensive way.

**Figure 12:**
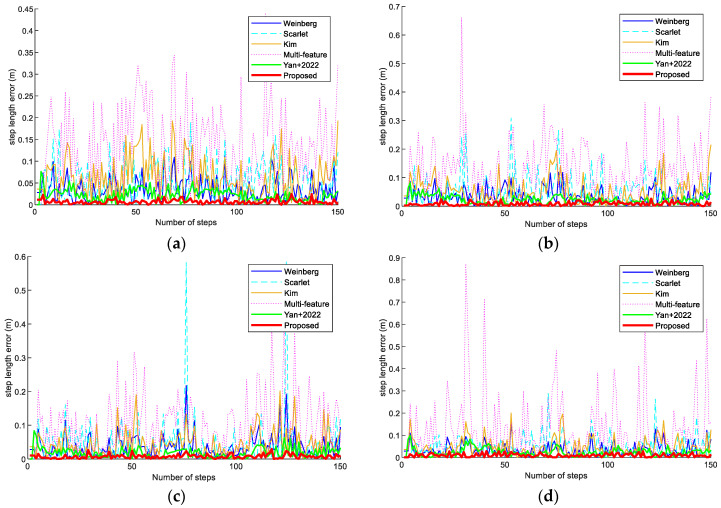
Step-length errors of the Weinberg, Scarlet, Kim, Multi-feature, Yan+ 2022 [26], and proposed step models in scene 2. (**a**) Volunteer #1 using an OPPO K5 smartphone. (**b**) Volunteer #1 using a Vivo X30 smartphone. (**c**) Volunteer #2 using an OPPO K5 smartphone. (**d**) Volunteer #2 using a Vivo X30 smartphone.

Figure 12 illustrates the step length errors of the Weinberg, Scarlet, Kim, Multi-feature, Yan+ 2022 [26], and proposed step models in scene 2. In longer paths, the step improvement model proposed in this paper had a smaller average step error and can achieve higher target localization. This is because the model estimation with constrained LASSO can obtain more features for a fine estimation.

Table 1 and Table 2 show the average step-length results among the Scarlet model, Kim model, Weinberg model, Multi-feature model, Yan+ 2022 [26] model, and proposed model for Volunteer #1 holding the OPPO K5 and Vivo X30 smartphones in the two scenes. The step estimation results of the proposed model have a higher step-length estimation performance for different scenarios and devices.

Table 3 and Table 4 show the average step-length estimation results of the Scarlet model, Kim model, Weinberg model, Multi-feature model, Yan+ 2022 [26] model, and the proposed model by Volunteer #2 holding OPPO K5 and Vivo X30 smartphones in the two scenes. The step estimation results demonstrate that the proposed model is more robust at different heights and has better universality.

### 4.3. CDF Positioning Performance

**Figure 13:**
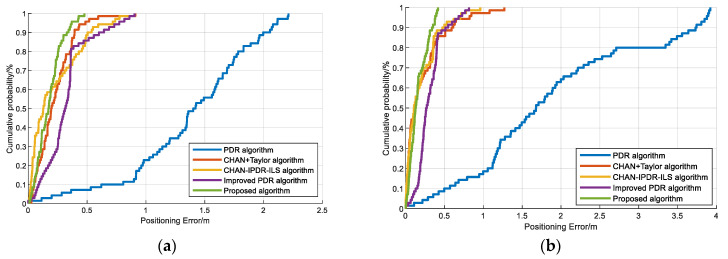

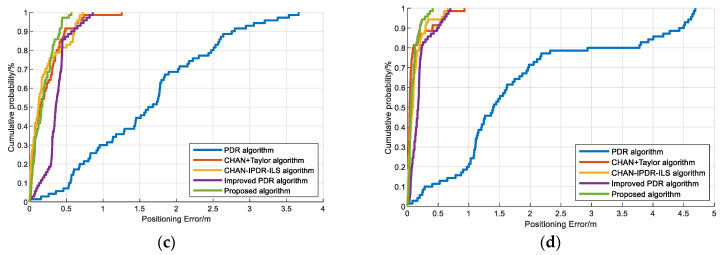
CDFs of the positioning errors on the different algorithms at the first scene: (**a**) Volunteer #1 using an OPPO K5 smartphone. (**b**) Volunteer #1 using a Vivo X30 smartphone. (**c**) Volunteer #2 using an OPPO K5 smartphone. (**d**) Volunteer #2 using a Vivo X30 smartphone.

To verify the positioning performance, we carried out multiple experiments on the PDR algorithm, CHAN–Taylor hybrid algorithm, CHAN–IPDR–ILS [34], improved PDR algorithm, and proposed algorithm using different phones in different scenarios. Figure 13 presents the localization performance of the above-mentioned algorithms for two volunteers using the Vivo X30 and OPPO K5 mobile phones in scene 1. The experiments show that the proposed algorithm has smaller positioning errors than the state-of-the-art algorithms. This method not only uses the weighted fusion step estimation model based on LASSO to improve the step accuracy of PDR but also combines it with acoustic estimation to reduce the cumulative error of PDR.

**Figure 14:**
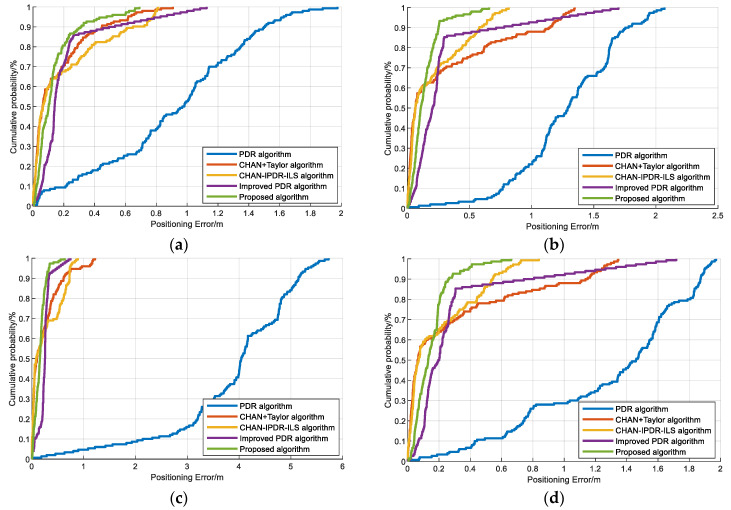
CDFs of the positioning errors on the different algorithms at the second scene: (**a**) Volunteer #1 using an OPPO K5 smartphone. (**b**) Volunteer #1 using a Vivo X30 smartphone. (**c**) Volunteer #2 using an OPPO K5 smartphone. (**d**) Volunteer #2 using a Vivo X30 smartphone.

Figure 14 presents the positioning performance of the PDR algorithm, CHAN–Taylor hybrid algorithm, CHAN–IPDR–ILS, improved PDR algorithm, and our algorithm by two volunteers using Vivo X30 and OPPO K5 smartphones in the second scene. The proposed algorithm has a smaller localization error over long movement times in similar scenes. The experiments demonstrate that the PDR algorithm in this paper significantly improves its positioning performance in similar scenarios, and the EKF fusion of the proposed positioning algorithm has the best positioning performance among these algorithms and solves the contradiction between high positioning accuracy and low cost. The main reason is that this method can extract accurate features for step-length prediction in the dead reckoning. The outlier schemes are determined during the fusion positioning process, and the EKF can achieve good nonlinear filtering.

**Figure 15:**
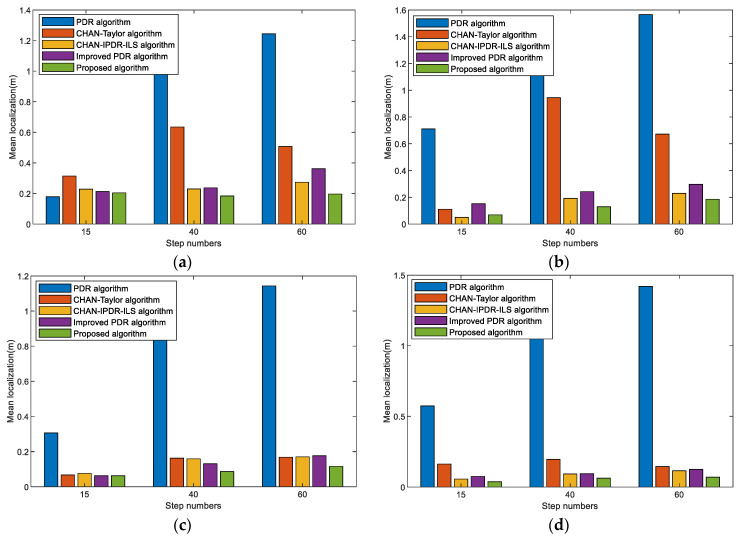
Mean localization errors of different step numbers on different algorithms in the first scene. (**a**) Volunteer #1 using an OPPO K5 smartphone. (**b**) Volunteer #1 using a Vivo X30 smartphone. (**c**) Volunteer #2 using an OPPO K5 smartphone. (**d**) Volunteer #2 using a Vivo X30 smartphone.

The mean localization errors of different step numbers for the PDR algorithm, CHAN–Taylor algorithm, CHAN–IPDR–ILS algorithm, improved PDR algorithm, and our algorithm at the first scene are shown in Figure 15. The proposed algorithm resulted in the least positioning errors for different length paths. This is because the method attenuates the cumulative error in the PDR algorithm over long movement times and the occasional error of the acoustic-based estimation.

**Figure 16:**
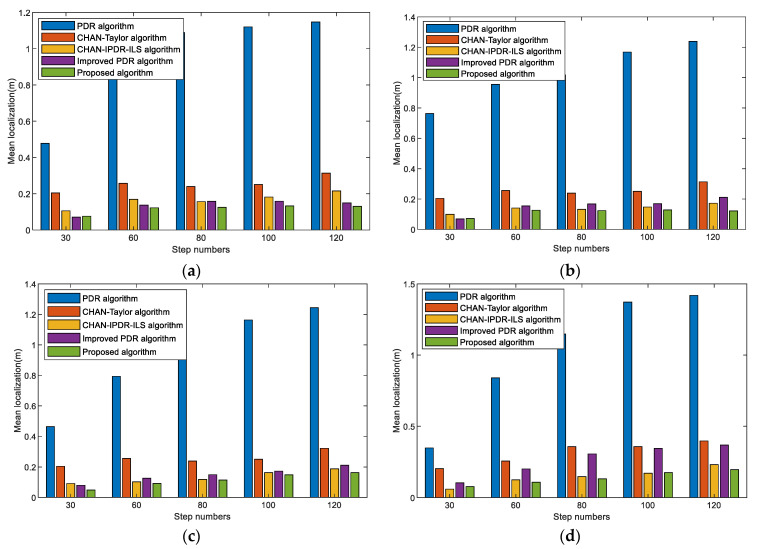
Mean localization errors of different step numbers on the different algorithms at the second scene. (**a**) Volunteer #1 using an OPPO K5 smartphone. (**b**) Volunteer #1 using a VivoX30 smartphone. (**c**) Volunteer #2 using an OPPO K5 smartphone. (**d**) Volunteer #2 using a Vivo X30 smartphone.

Figure 16 shows the mean localization errors of different length paths in different algorithms with different smartphones and pedestrians in the second scene. The results reveal that the positioning errors of the proposed algorithm increase slightly as the step numbers increases. However, the overall positioning performance remains basically stable, and the accumulated errors are effectively reduced. The improved PDR has better performance in the cumulative errors. The proposed system exhibits a good positioning performance in different length paths, and good robustness and universality. This is because device heterogeneity and pedestrian step differences during step-length prediction are effectively eliminated, and pedestrian motion features are accurately extracted. In addition, the impact of the environment on acoustic signal localization is addressed.

### 4.4. Mean and RMS Error Performance

Table 5 and Table 6 present the mean and RMS errors among the different algorithms in scene 1. The PDR algorithm in this paper has good robustness for different pedestrians. Due to equipment heterogeneity, the proposed algorithm has slightly different errors, of less than 10 cm. In this open symmetric scene, the proposed positioning system has better performance than the other algorithms. This is because the improved PDR algorithm effectively addresses the accumulated errors, and EKF fusion reduces the nonlinearity effect.

Table 7 and Table 8 describe the mean and RMS errors in the different algorithms in scene 2. The proposed algorithm has better performance than the others. Moreover, it can be revealed that the proposed EKF-based PDR method combined with acoustic estimation has better localization accuracy, and more robust localization performance under many different experimental conditions. Its greatest highlight is that the step-length estimation model is based on LASSO. Therefore, more accurate localization can be achieved.

## 5. Conclusions

In this paper, we present a localization method that utilizes the EKF to fuse acoustic estimation and improve PDR. In the method in this paper, acoustic estimation is implemented using a hybrid CHAN–Taylor algorithm. In the dead reckoning, we propose a novel weighted step-length model with constraint LASSO. In this model, the peak and valley of the current step and the previous three steps are used to obtain a coarse step estimation; then, acceleration peak-to-valley amplitude difference, walk frequency, variance of acceleration, mean acceleration, peak median, valley median are extracted from the collected data. Finally, we combine the extracted motion features and LASSO regularization spatial constraints to obtain accurate step length. The model utilizes LASSO regression to combine multiple features to predict step results and improve the step estimation accuracy of PDR. Finally, the improved PDR is used as the state model and the acoustic estimation is used as the observation model. The target location is determined by EKF fusion.

To demonstrate the localization accuracy of the proposed method, we conducted extensive experiments on different experimental paths in two scenes. Scene 1 is a reading room with an area of approximately 432 m^2^, and scene 2 is a corridor with an area of approximately 584.8 m^2^. Two volunteers with different heights holding Vivo X30 and OPPO K5 smartphones were recruited to collect the data in the experiment. The experimental results demonstrate that the proposed step-length method is more accurate at comparing than the Weinberg, Scarlet, Kim, Multi-feature, Yan+ 2022 [26] model. It validates that our method can extract more accurate information to achieve high performance. Finally, we fuse the acoustic positioning with dead reckoning to obtain high positioning performance and low lost. Different pedestrians and devices were carried out in two different scenes. The experimental results show that the proposed positioning system can achieve a more accurate localization performance in the case of different users and different devices. The localization method can effectively mitigate the cumulative error of PDR and improve the accuracy and stability of indoor positioning. Although we conducted the experiment in different scenes, there were not enough obstacles for the two scenes. Therefore, the underground parking lot is an interesting test scenario for future work.

## Figures and Tables

**Table 1:** Estimation of the average step length in scene 1 by Volunteer #1 using the OPPO K5 and Vivo X30 smartphone.

Device	Method	Average Step Length (m)
OPPO K5	Scarlet model	0.6265
	Kim model	0.5576
	Weinberg model	0.5688
	Multi-feature model	0.5756
	Yan+ 2022 [26] model	0.5913
	Proposed model	0.6013
Vivo X30	Scarlet model	0.6201
	Kim model	0.5527
	Weinberg model	0.5635
	Multi-feature model	0.5838
	Yan+ 2022 [26] model	0.5877
	Proposed model	0.5991

**Table 2:** Estimation of the average step length in scene 2 by Volunteer #1 using the OPPO K5 and Vivo X30 smartphone.

Device	Method	Average Step Length (m)
OPPO K5	Scarlet model	0.6306
	Kim model	0.5606
	Weinberg model	0.5814
	Multi-feature model	0.5875
	Yan+ 2022 [26] model	0.5916
	Proposed model	0.6010
Vivo X30	Scarlet model	0.6263
	Kim model	0.5564
	Weinberg model	0.5797
	Multi-feature model	0.5859
	Yan+ 2022 [26] model	0.5907
	Proposed model	0.5992

**Table 3:** Estimation of the average step length in scene 1 by Volunteer #2 using the OPPO K5 and Vivo X30 smartphone.

Device	Method	Average Step Length (m)
OPPO K5	Scarlet model	0.6337
	Kim model	0.5631
	Weinberg model	0.5765
	Multi-feature model	0.5775
	Yan+ 2022 [26] model	0.6114
	Proposed model	0.6056
Vivo X30	Scarlet model	0.6298
	Kim model	0.5658
	Weinberg model	0.5649
	Multi-feature model	0.5755
	Yan+ 2022 [26] model	0.5900
	Proposed model	0.6005

**Table 4:** Estimation of the average step length in scene 2 by Volunteer #2 using the OPPO K5 and Vivo X30 smartphone.

Device	Method	Average Step Length (m)
OPPO K5	Scarlet model	0.6297
	Kim model	0.5630
	Weinberg model	0.5813
	Multi-feature model	0.5824
	Yan+ 2022 [26] model	0.6095
	Proposed model	0.5996
Vivo X30	Scarlet model	0.6243
	Kim model	0.5462
	Weinberg model	0.5880
	Multi-feature model	0.5878
	Yan+ 2022 [26] model	0.5894
	Proposed model	0.5995

**Table 5:** Mean and RMS error comparison among different algorithms for Volunteer #1 holding the OPPO K5 and Vivo X30 in scene 1 (m).

Device	Method	Mean Error	RMS Error
OPPO K5	PDR algorithm	1.3795	1.4714
	CHAN–Taylor algorithm	0.2339	0.2706
	CHAN–IPDR–ILS algorithm	0.2219	0.3126
	Improved PDR algorithm	0.3249	0.3780
	Proposed algorithm	0.1640	0.1881
Vivo X30	PDR algorithm	1.8636	2.3054
	CHAN–Taylor algorithm	0.1101	0.2127
	CHAN–IPDR–ILS algorithm	0.1229	0.1905
	Improved PDR algorithm	0.2009	0.2578
	Proposed algorithm	0.0905	0.1237

**Table 6:** Mean and RMS error comparison among different algorithms for Volunteer #2 holding the OPPO K5 and Vivo X30 in scene 1 (m).

Device	Method	Mean Error	RMS Error
OPPO K5	PDR algorithm	1.6189	1.8466
	CHAN–Taylor algorithm	0.2217	0.3332
	CHAN–IPDR–ILS algorithm	0.2034	0.3004
	Improved PDR algorithm	0.3688	0.4056
	Proposed algorithm	0.1804	0.3004
Vivo X30	PDR algorithm	1.8835	2.1757
	CHAN–Taylor algorithm	0.2224	0.3285
	CHAN–IPDR–ILS algorithm	0.1955	0.2877
	Improved PDR algorithm	0.3078	0.3490
	Proposed algorithm	0.1674	0.2072

**Table 7:** Mean and RMS error comparison among different algorithms for Volunteer #1 holding the OPPO K5 and Vivo X30 in scene 2 (m).

Device	Method	Mean Error	RMS Error
OPPO K5	PDR algorithm	0.9076	1.0232
	CHAN–Taylor algorithm	0.1583	0.3184
	CHAN–IPDR–ILS algorithm	0.1983	0.2536
	Improved PDR algorithm	0.2123	0.3111
	Proposed algorithm	0.1395	0.1981
Vivo X30	PDR algorithm	1.4852	1.6446
	CHAN–Taylor algorithm	0.1637	0.2819
	CHAN–IPDR–ILS algorithm	0.1205	0.2010
	Improved PDR algorithm	0.1474	0.2003
	Proposed algorithm	0.1188	0.1200

**Table 8:** Mean and RMS error comparison among different algorithms for Volunteer #2 holding the OPPO K5 and Vivo X30 in scene 2 (m).

Device	Method	Mean Error	RMS Error
OPPO K5	PDR algorithm	3.8788	4.0600
	CHAN–Taylor algorithm	0.2356	0.3685
	CHAN–IPDR–ILS algorithm	0.2528	0.3782
	Improved PDR algorithm	0.2461	0.2754
	Proposed algorithm	0.1623	0.1900
Vivo X30	PDR algorithm	2.4835	2.7227
	CHAN–Taylor algorithm	0.2146	0.3798
	CHAN–IPDR–ILS algorithm	0.1718	0.2706
	Improved PDR algorithm	0.2063	0.2746
	Proposed algorithm	0.1489	0.1879

## Data Availability

All test data mentioned in this paper will be made available on request to the corresponding author’s email with appropriate justification.
